# Perceived COVID-19 vaccine effectiveness, acceptance, and drivers of vaccination decision-making among the general adult population: A global survey of 20 countries

**DOI:** 10.1371/journal.pntd.0010103

**Published:** 2022-01-28

**Authors:** Roy Rillera Marzo, Absar Ahmad, Md. Saiful Islam, Mohammad Yasir Essar, Petra Heidler, Isabel King, Arulmani Thiyagarajan, Kittisak Jermsittiparsert, Karnjana Songwathana, Delan Ameen Younus, Radwa Abdullah El-Abasiri, Burcu Kucuk Bicer, Nhat Tan Pham, Titik Respati, Susan Fitriyana, Erwin Martinez Faller, Aries Moralidad Baldonado, Md Arif Billah, Yadanar Aung, Shehu Muhammad Hassan, Muhammad Mujtaba Asad, Kareem Ahmed El-Fass, Sudip Bhattacharya, Sunil Shrestha, Nouran Ameen Elsayed Hamza, Pascal Friedmann, Michael Head, Yulan Lin, Siyan Yi

**Affiliations:** 1 Department of Community Medicine, International Medical School, Management and Science University, Shah Alam, Selangor Darul Ehsan, Malaysia; 2 Department of Community Medicine, Faculty of Medicine, Asia Metropolitan University, Johor Bahru, Malaysia; 3 Global Public Health, Jeffrey Cheah School of Medicine and Health Sciences, Monash University Malaysia, Bandar Sunway, Malaysia; 4 Department of Community Medicine, Manipal Tata Medical College, Jamshedpur, Manipal Academy of Higher Education, Manipal, India; 5 Department of Public Health and Informatics, Jahangirnagar University, Savar, Dhaka, Bangladesh; 6 Centre for Advanced Research Excellence in Public Health, Savar, Dhaka, Bangladesh; 7 Kabul University of Medical Sciences, Kabul, Afghanistan; 8 Department of Health Sciences, St. Pölten University of Applied Sciences, St. Pölten, Austria; 9 Department of International Business and Export Management, IMC University of Applied Sciences Krems, Krems an der Donau, Austria; 10 University for Continuing Education Krems, Department for Economy and Health, Krems, Austria; 11 University of the Sunshine Coast, School of Health and Behavioural Sciences, Maroochydore, Australia; 12 Sunshine Coast Health Institute, Sunshine Coast University Hospital and Health Service, Birtinya, Australia; 13 Department of Clinical Epidemiology, Leibniz Institute for Prevention Research and Epidemiology—BIPS, Bremen, Germany; 14 College of Innovative Business and Accountancy, Dhurakij Pundit University, Bangkok, Thailand; 15 Faculty of Economics and Investment, Bangkok University, Bangkok, Thailand; 16 General Directorate for Scientific Research Center, Salahaddin University-Erbil, Erbil, Iraq; 17 Nuffield Department of Population Health, University of Oxford, Oxford, United Kingdom; 18 Faculty of Medicine, Department of Medical Education and Informatics, Gazi University, Ankara, Turkey; 19 School of Business, International University, Ho Chi Minh City, Vietnam; 20 Vietnam National University, Ho Chi Minh City, Vietnam; 21 Department of Public Health, Faculty of Medicine and Faculty of Graduate Studies, Universitas Islam Bandung, Bandung, Indonesia; 22 Faculty of Medicine, Universitas Islam Bandung, Bandung, Indonesia; 23 Department of Pharmacy, Faculty of Pharmacy, San Pedro College, Davao City, Philippines; 24 College of Nursing, Saint Alexius College, City of Koronadal, Philippines; 25 Faculty of Business, Economics and Social Development, Universiti Malaysia Terengganu, Kuala Terengganu, Malaysia; 26 Medical Statistics Division, Department of Medical Research, Pyin Oo Lwin, Myanmar; 27 Institute for Population and Social Research, Mahidol University, Nakhon Pathom, Thailand; 28 Faculty of Life Sciences, Ahmadu Bello University, Zaria, Nigeria; 29 Education Department, Sukkur IBA University, Sukkur, Pakistan; 30 Department of Pharmacy Practice, College of Clinical Pharmacy, King Faisal University, Al Hassa, Saudi Arabia; 31 Department of Community Medicine, Himalayan Institute of Medical Sciences, Dehradun, India; 32 School of Pharmacy, Monash University Malaysia, Bandar Sunway, Malaysia; 33 Medical Agency for Research and Statistics, Giza, Egypt; 34 Clinical Research Key (CRK-CRO), Nairobi, Kenya; 35 Lumpkin College of Business and Technology, Eastern Illinois University, Charleston, Illinois, United States of America; 36 Clinical Informatics Research Unit, Faculty of Medicine, University of Southampton, Southampton, United Kingdom; 37 Department of Epidemiology and Health Statistics, School of Public Health, Fujian Medical University, Fuzhou, China; 38 Saw Swee Hock School of Public Health, National University of Singapore and National University Health System, Singapore, Singapore; 39 KHANA Center for Population Health Research, Phnom Penh, Cambodia; 40 Center for Global Health Research, Touro University California, Vallejo, California, United States of America; KU Leuven, BELGIUM

## Abstract

**Background:**

Mass vaccination campaigns have significantly reduced the COVID-19 burden. However, vaccine hesitancy has posed significant global concerns. The purpose of this study was to determine the characteristics that influence perceptions of COVID-19 vaccine efficacy, acceptability, hesitancy and decision making to take vaccine among general adult populations in a variety of socioeconomic and cultural contexts.

**Methods:**

Using a snowball sampling approach, we conducted an online cross-sectional study in 20 countries across four continents from February to May 2021.

**Results:**

A total of 10,477 participants were included in the analyses with a mean age of 36±14.3 years. The findings revealed the prevalence of perceptions towards COVID-19 vaccine’s effectiveness (78.8%), acceptance (81.8%), hesitancy (47.2%), and drivers of vaccination decision-making (convenience [73.3%], health providers’ advice [81.8%], and costs [57.0%]). The county-wise distribution included effectiveness (67.8–95.9%; 67.8% in Egypt to 95.9% in Malaysia), acceptance (64.7–96.0%; 64.7% in Australia to 96.0% in Malaysia), hesitancy (31.5–86.0%; 31.5% in Egypt to 86.0% in Vietnam), convenience (49.7–95.7%; 49.7% in Austria to 95.7% in Malaysia), advice (66.1–97.3%; 66.1% in Austria to 97.3% in Malaysia), and costs (16.0–91.3%; 16.0% in Vietnam to 91.3% in Malaysia). In multivariable regression analysis, several socio-demographic characteristics were identified as associated factors of outcome variables including, i) vaccine effectiveness: younger age, male, urban residence, higher education, and higher income; ii) acceptance: younger age, male, urban residence, higher education, married, and higher income; and iii) hesitancy: male, higher education, employed, unmarried, and lower income. Likewise, the factors associated with vaccination decision-making including i) convenience: younger age, urban residence, higher education, married, and lower income; ii) advice: younger age, urban residence, higher education, unemployed/student, married, and medium income; and iii) costs: younger age, higher education, unemployed/student, and lower income.

**Conclusions:**

Most participants believed that vaccination would effectively control and prevent COVID-19, and they would take vaccinations upon availability. Determinant factors found in this study are critical and should be considered as essential elements in developing COVID-19 vaccination campaigns to boost vaccination uptake in the populations.

## Author summary

This large-scale multi-country study explores perceived COVID-19 vaccine effectiveness, acceptability, and hesitancy rates and their related factors among the general adult populations from 20 countries. We found that a considerable proportion of the participants has an intention to receive the COVID-19 vaccine. However, more than half still expressed their hesitancy to receive the vaccines. Countries where the majority of respondents responded that they would accept COVID-19 vaccination included Malaysia (96.0%), Bangladesh (93.6%), and Iraq (91.8%). The majority of the participants from Vietnam (86.0%) and Turkey (74.7%) expressed hesitance to receive COVID-19 vaccine. Perceived vaccine effectiveness ranged from 67.8% in Egypt to 95.9% in Malaysia. The participants believed that convenience, health providers’ advice, and costs of vaccines are important for people to decide whether to accept COVID-19 vaccines. Country of residence, age, sex, level of education, area of residence, marital status, and family economic status were significantly associated with tendency to undergo COVID-19 vaccination and the perception that COVID-19 vaccine decision-making is influenced by convenience. The findings of this study provide useful guidance for tailored interventions to enhance the acceptance of COVID-19 vaccine. Promotional program addressed on detected socioeconomic factors is warranted.

## Introduction

The global COVID-19 pandemic has raised imminent public health threats to people across the globe. As it continues spreading, the burden of the cases has become more worrisome. As of September 4, 2021, over 219 million cases have been recorded globally [1]. The epidemic has hit low- and middle-income nations due to relatively weak health care systems in responding to such a public health emergency. The fight against COVID-19 has taken various trends in different parts of the world, with some countries bracing against the third wave of the pandemic [2–5]. South Asian countries like India have experienced colossal impacts of COVID-19, such as oxygen shortage and Delta variant that have exploded the healthcare system in the country [6,7]. Mass vaccination has started to beat the deadly pandemic, but more vaccines are required to supply the large population [8,9]. To prevent further spread, governments worldwide have adopted several prevention measures to contain the pandemic in its early days. However, lessons from the past have revealed that the best tool in preventing any viral epidemic and pandemic is safe and secure vaccines [10].

The World Health Organization (WHO) has approved several vaccines for emergency use against the COVID-19 pandemic. Mass vaccination campaigns across the globe have significantly reduced the burden of cases. However, along with the movements, adverse drug reactions of vaccines, acceptance of vaccines, and hesitancy in populations have raised severe concerns [11,12] and may pose a significant global health threat. Vaccine hesitancy is defined as an individual’s unwillingness to take the vaccines despite their availability [13]. Primary reasons for the willingness to accept vaccination include convenience, complacency, and confidence [14]. Convenience comprises the availability, affordability, and vaccines delivery in a comfortable context. Complacency underscores the low disease risk perception; thus, the need for immunization was deemed unnecessary. Confidence denotes the trust in vaccination safety, effectiveness, and healthcare systems competence [15].

The impact of vaccine hesitancy is threatening the world as it has been listed in the top ten global health threats in 2019 [16,17]. The past rises in measles cases have been linked to vaccine hesitancy as one of the known reasons [18]. To this day, vaccine hesitancy has remained a significant challenge to the world. The COVID-19 pandemic has further reignited the notion of vaccine hesitancy. The rapid vaccine development and media coverage of misinformation are among the reasons behind the rationale of hesitancy towards vaccination [11,19].

Amid the COVID-19 pandemic, the hesitancy in vaccination has taken a surge across the world. This plight has plagued many countries and slowed down the scale of vaccination coverage. To date, many studies have been conducted in different countries and populations to address these concerns [20–24]. Afghanistan, a low-income country with low vaccination coverage, has also witnessed a trend in the hesitancy towards vaccination. Nemat et al. have revealed that over 37% of the population from different provinces in Afghanistan expressed reluctance to get the COVID-19 vaccination [25]. Primary reasons for the unwillingness to get the vaccines were a perception of low vaccine quality and safety and a belief that they have sufficient immunity to combat COVID-19 [25].

A population-based survey in Nigeria during the pandemic peak indicated a high willingness to get the COVOD-19 vaccines among the general public [26]. However, 25% of the participants expressed their hesitancy [26]. The primary reasons behind the hesitancy included the perceived unreliability of clinical trials, a belief that they have strong immunity to fight against the disease, and a concern over vaccine safety [26]. These findings emphasize the need for more strategic interventions to understand the barriers better and increase vaccine acceptance across the world.

However, there is a lack of multi-country population-based studies involving different socioeconomic and cultural contexts. A previous study conducted in nine lower- and middle-income countries (LIMCs) reported only COVID-19 prevalence and factors related to vaccine acceptance. Therefore, this large-scale multi-country study explored perceived COVID-19 vaccine effectiveness, acceptability, and hesitancy rates and their related factors among the general adult populations in different socioeconomic and cultural contexts. This study findings will help countries plan for better control of vaccination programs and better prepare for future pandemics.

## Methods

### Ethics statement

The study was designed and conducted in line with the declaration of Helsinki and was approved by the Asia Metropolitan University Ethics Committee in Malaysia (Ref. No: AMU/MREC/NF/28032020). Respondents were informed that their participation was voluntary, and written consent was implied on the completion of the questionnaire. All participants were aged 18 years or older.

### Study design and sampling

An online cross-sectional study was conducted using a google form in Australia, Austria, Bangladesh, Denmark, Egypt, Germany, India, Indonesia, Iraq, Ireland, Malaysia, Myanmar, New Zealand, Nigeria, the Philippines, Scotland, Sri Lanka, Thailand, Turkey, and Vietnam. Data were collected between February 2021 and May 2021 using a snowball sampling approach.

### Data collection procedures

We distributed the questionnaire using personal contacts by emails, web-based applications (e.g., WhatsApp, Telegram), and social media (e.g., Facebook, LinkedIn, Twitter, and Instagram). Participants must be aged 18 years or older. They were reminded to respond only once and use a unique identifier to create a single account by settings that allow one response per user. Finally, the confidentiality and privacy of participants’ responses were ensured to minimize potential bias caused by self-reported data.

Data collected using google forms offered an excel format to survey output to analyze the raw data offline. All country’s data in excel were exported to IBM SPSS (version 25). De-identifications were done to secure the safety of respondents, which removed the identifiers like name, email ID, or mobile number that directly or indirectly point to a person.

### Tool development and measures

We developed the questionnaire through participatory discussion within the research team of participating countries. The COVID-19 related questions were adapted from a previous study [27] conducted on 18 years and above. The questionnaire was completed in less than five minutes. The questionnaire was first designed in English and then translated into the participant countries native languages. It was created to alleviate responder fatigue and was face-validated by experts.

Sociodemographic characteristics of the participants included age (continuous), sex (male/female), place of residence (rural/urban), education (secondary or less/post-secondary/tertiary), employment status (employed/student/unemployed), marital status (ever married/never married), and perceived family economic status (low/medium/high).

Participants’ perception of vaccine effectiveness was assessed by asking whether they agreed that vaccination effectively prevents and controls COVID-19. COVID-19 vaccine acceptance was assessed by asking if the participant would like to get COVID-19 vaccines if they are successfully produced and authorized for use in the future. We also asked whether the participant agreed that convenience, health providers’ advice, and vaccine costs are important factors for deciding whether they should receive the vaccines. To measure vaccine hesitancy, participants were asked whether they would get the vaccine as soon as possible if it become available. The responses of all the questions were binary (yes/no).

### Statistical analyses

For continuous variables, mean and standard deviation (SD) were calculated. Categorical variables were presented using percentages. In bivariate analyses, we used the Chi-square test to explore associations between categorical variables. Multivariable logistic regression models were constructed to explore factors associated with perceived vaccine effectiveness, acceptance, and drivers of COVID-19 vaccine uptake. The socio-demographic and economic characteristics of the participants were simultaneously included in each model. Adjusted odds ratio (AOR) with 95% confidence intervals (95% CI) were reported. Statistical significance was considered at *p*<0.05.

## Results

A total of 11,024 participants responded to the survey including 547 incomplete responses. In the final analysis, 10,477 participants were included with a mean age 36 years (SD = 14.28). More than two-thirds (70.8%) were urban residents, 56.7% were female, 47.6% had tertiary education, 63.9% were employed, 53.5% were ever married, and 47.1% reported medium family economic status (Table 1). Thailand had the most significant number of participants (22.6%), followed by Austria (12.9%), Malaysia (12.2%), and Iraq (9.7%).

**Table 1:** Study sample characteristics according to residence country (*n* = 10,477).

	AU	AT	BD	EG	ID	IQ	MY	MM	NG	PH	TH	TR	VN	Other	Total
Number	156	1347	359	957	522	1021	1283	301	201	313	2365	827	651	174	10477
Age															
<30 years	22(14.1)	188(14.0)	259(72.1)	723(75.5)	329(63.0)	311(30.5)	964(75.1)	98(32.6)	102(50.7)	228(72.8)	791(33.4)	261(31.6)	395(60.7)	42(24.1)	4713(45.0)
31–45 years	51(32.7)	500(37.1)	48(13.4)	186(19.4)	120(23.0)	327(32.0)	229(17.8)	174(57.8)	90(44.8)	57(18.2)	939(39.7)	140(16.9)	237(36.4)	75(43.1)	3173(30.3)
46–60 years	42(26.9)	521(38.7)	36(10.0)	40(4.2)	63(12.1)	242(23.7)	71(5.5)	21(7.0)	7(3.5)	22(7.0)	579(24.5)	232(28.1)	19(2.9)	50(28.7)	1945(18.6)
>60 years	41(26.3)	138(10.2)	16(4.5)	8(0.8)	10(1.9)	141(13.8)	19(1.5)	8(2.7)	2(1.0)	6(1.9)	56(2.4)	194(23.5)	0(0.0)	7(4.0)	646(6.2)
Female	92 (59.0)	1106 (82.1)	180 (50.1)	598 (62.5)	376 (72.0)	530 (51.9)	371 (28.9)	176 (58.5)	59 (29.4)	247 (78.9)	1288 (54.5)	453 (54.8)	340 (52.2)	121 (69.5)	5937 (56.7)
Urban resident	94 (60.3)	786 (58.4)	213 (59.3)	751 (78.5)	310 (59.4)	916 (89.7)	1146 (89.3)	269 (89.4)	154 (76.6)	166 (53.0)	1602 (67.7)	687 (83.1)	199 (30.6)	123 (70.7)	7416 (70.8)
Education level															
<Secondary	58 (37.2)	483 (35.9)	33 (9.2)	23 (2.4)	278 (53.3)	471 (46.1)	67 (5.2)	4 (1.3)	8 (4.0)	20 (6.4)	492 (20.8)	540 (65.3)	105 (16.1)	37 (21.3)	2619 (25.0)
Secondary	98 (62.8)	169 (12.5)	58 (16.2)	500 (52.2)	244 (46.7)	424 (41.5)	284 (22.1)	42 (14.0)	33 (16.4)	0	705 (29.8)	0	262 (40.2)	51 (29.3)	2870 (27.4)
>Secondary	0	695 (51.6)	268 (74.7)	434 (45.4)	0	126 (12.3)	932 (72.6)	255 (84.7)	160 (79.6)	293 (93.6)	1168 (49.4)	287 (34.7)	284 (43.6)	86 (49.4)	4988 (47.6)
Currently employed	134 (85.9)	1239 (92.0)	126 (35.1)	682 (71.3)	188 (36.0)	566 (55.4)	377 (29.4)	261 (86.7)	111 (55.2)	126 (40.3)	1924 (81.4)	383 (46.3)	433 (66.5)	144 (82.8)	6694 (63.9)
Ever married	118 (75.6)	1080 (80.2)	154 (42.9)	325 (34.0)	269 (51.5)	686 (67.2)	328 (25.6)	153 (50.8)	95 (47.3)	75 (24.0)	1342 (56.7)	511 (61.8)	345 (53.0)	124 (71.3)	5605 (53.5)
Family economic status															
Low	27 (17.3)	195 (14.5)	92 (25.6)	122 (12.7)	286 (54.80	290 (28.4)	366 (28.5)	44 (14.6)	26 (12.9)	119 (38.0)	1045 (44.2)	247 (29.9)	137 (21.0)	20 (11.5)	3016 (28.8)
Medium	68 (43.6)	761 (56.5)	138 (38.4)	337 (35.2)	221 (42.3)	557 (54.6)	831 (64.8)	75 (24.9)	18 (9.0)	193 (61.7)	905 (38.3)	413 (49.9)	335 (51.5)	81 (46.6)	4933 (47.1)
High	61 (39.1)	391 (29.0)	129 (35.9)	498 (52.0)	15 (2.9)	174 (17.0)	86 (6.7)	182 (60.5)	157 (78.1)	1 (0.3)	415 (17.5)	167 (20.2)	179 (27.5)	73 (42.0)	2528 (24.1)

Values are the number (%) for categorical variables and mean (SD) for continuous variables. AU: Australia, AT: Austria, BD: Bangladesh, EG: Egypt, ID: Indonesia, IQ: Iraq, MY: Malaysia, MM: Myanmar, NG: Nigeria, PH: Philippines, TH: Thailand, TR: Turkey, VN: Vietnam. Other countries include Canada, China, Germany, India, Ireland, New Zeeland, Scotland, Sri-Lanka, and the United Kingdom.

As shown in Table 2, 78.8% of the participants agreed that vaccination would effectively control and prevent COVID-19, and 81.8% would get COVID-19 vaccines if they are successfully produced and authorized for use in the future. The participants believed that convenience (73.3%), health providers’ advice (81.8%), and costs of vaccines (57.0%) are important for people to decide whether to accept COVID-19 vaccines. About half (52.8%) expressed their hesitancy to receive the vaccines.

**Table 2:** Perceived effectiveness, acceptance, and drivers of COVID-19 vaccine uptake decision-making by country by socio-demographic characteristics (*n* = 10,477).

Socio-demographic characteristics		Agreed that vaccines are effective to prevent and control COVID-19	Would accept COVID-19 vaccines when available	Believed that convenience is important for people to decide whether to accept vaccines	Believed that health providers’ advice is important for people to decide whether to accept vaccines	Believed that cost of the vaccines is important for people to decide whether to accept vaccines	Expressed hesitancy to receive COVID-19 vaccines
Age group							
	<30 years	3975 (84.3)	4114 (87.3)	3858 (81.9)	4196 (89.0)	3195 (67.8)	2372 (50.3)
	31 to 45	2392 (75.4)	2505 (78.9)	2109 (66.5)	2441 (76.9)	1587 (50.0)	1680 (52.1)
	46 to 60	1372 (70.5)	1430 (73.5)	1230 (63.2)	1399 (71.9)	881 (45.3)	1064 (54.7)
	60+	514 (79.6)	523 (81.0)	479 (74.1)	538 (83.3)	313 (48.5)	419 (64.9)
		P < 0.001**	P < 0.001**	P < 0.001**	P < 0.001**	P < 0.001**	P < 0.001**
Female		4482 (75.5)	4682 (78.9)	4151 (69.9)	4727 (79.6)	3175 (53.5)	3112 (52.4)
		P < 0.001**	P < 0.001**	P < 0.001**	P < 0.001**	P < 0.001**	P = 0.33
Urban residence		6022 (81.2)	6300 (85.0)	5733 (77.3)	6271 (84.6)	4492 (60.6)	3840 (51.8)
		P < 0.001**	P < 0.001**	P < 0.001**	P < 0.001**	P < 0.001**	P < 0.001**
Level of education							
	< Secondary	1864 (71.2)	1861 (71.1)	1685 (64.3)	1936 (73.9)	1230 (47.0)	1546 (59.0)
	Secondary	2239 (78.0)	2281 (79.5)	2107 (73.4)	2265 (78.9)	1611 (56.1)	1471 (51.3)
	> Secondary	4150 (83.2)	4430 (88.8)	3884 (77.9)	4373 (87.7)	3135 (62.9)	2518 (50.5)
		P < 0.001**	P < 0.001**	P < 0.001**	P < 0.001**	P < 0.001**	P < 0.001**
Currently employed		5064 (75.6)	5346 (79.9)	4593 (68.6)	5226 (78.1)	3388 (50.6)	3607 (53.9)
		P < 0.001**	P < 0.001**	P < 0.001**	P < 0.001**	P < 0.001**	P = 0.004**
Ever married		4291 (76.6)	4472 (79.8)	3767 (67.2)	4426 (79.0)	2726 (48.6)	3079 (54.9)
		P < 0.001**	P < 0.001**	P < 0.001**	P < 0.001**	P < 0.001**	P < 0.001**
Family economic status							
	Low	2285 (75.8)	2360 (78.2)	2242 (74.3)	2436 (80.8)	1924 (63.8)	1624 (53.8)
	Medium	3924 (79.5)	4035 (81.8)	3607 (73.1)	4063 (82.4)	2789 (56.5)	2566 (52.0)
	High	2044 (80.9)	2177 (86.1)	1827 (72.3)	2075 (82.1)	1263 (50.0)	1345 (53.2)
		P < 0.001**	P < 0.001**	P < 0.001**	P < 0.001**	P < 0.001**	P < 0.26
Total		8253 (78.8)	8572 (81.8)	7676 (73.3)	8574 (81.8)	5976 (57.0)	5535 (52.8)

** Significant at P <0.01

Table 3 shows that 95.9% of the participants from Malaysia thought that vaccination would effectively prevent and control COVID-19, followed by those from the Philippines (88.8%) and Vietnam (88.3%). Countries where majority of respondents responded that they would accept COVID-19 vaccination included Malaysia (96.0%), Bangladesh (93.6%), and Iraq (91.8%). Regarding perceived drivers of vaccine uptake decision-making, 95.7% of the participants from Malaysia, 93.0% from the Philippines, and 90.8% from Bangladesh agreed that convenience is an important factor for deciding whether they would receive the vaccines. Majority of the participants from Malaysia (97.3%), the Philippines (93.9%), and Indonesia (93.5%) agreed that health providers’ advice is important in people’s decision-making to get the COVID-19 vaccines. Vaccine costs were also believed to be an important factor among the participants from Malaysia (91.3%), the Philippines (81.5%), and Turkey (78.4%). Majority of the participants from Vietnam (86.0%) and Turkey (74.7%) expressed hesitance to receive COVID-19 vaccines.

**Table 3:** Perceived COVID-19 vaccine effectiveness, acceptance, and determinants of the uptake decision-making by country (*n* = 10,477).

Country	Agreed that vaccines are effective to prevent and control COVID-19,% (95%CI)	Would accept COVID-19 vaccines when available, % (95%CI)	Believed that convenience is important for people to decide whether to accept vaccines, % (95%CI)	Believed that health providers’ advice is important for people to decide whether to accept vaccines, % (95%CI)	Believed that cost of the vaccines is important for people to decide whether to accept vaccines, % (95%CI)	Expressed hesitancy to receive COVID-19 vaccines, % (95%CI)
Australia	84.0 (78.0–90.0)	64.7 (57.2–72.2)	71.8 (64.7–78.9)	78.8 (72.4–85.2)	55.8 (48.0–63.5)	53.2 (45.4–61.0)
Austria	70.4 (67.9–72.8)	71.0 (68.6–73.5)	49.7 (47.0–52.3)	66.1 (63.5–68.6)	22.6 (20.4–24.9)	65.3 (62.7–67.8)
Bangladesh	83.0 (79.1–86.9)	93.6 (91.0–96.1)	90.8 (87.8–93.8)	92.5 (89.7–95.2)	73.3 (68.7–77.8)	52.9 (47.8–58.1)
Egypt	67.8 (64.9–70.8)	80.8 (78.3–83.3)	85.7 (83.5–87.9)	87.0 (84.9–89.2)	75.3 (72.6–78.1)	31.5 (28.5–34.4)
Indonesia	86.0 (83.0–89.0)	68.6 (64.6–72.6)	64.6 (60.4–68.7)	93.5 (91.4–95.6)	42.7 (38.5–47.0)	68.6 (64.6–72.6)
Iraq	84.4 (82.2–86.6)	91.8 (90.1–93.5)	79.5 (77.0–82.0)	87.7 (85.6–89.7)	36.0 (33.1–39.0)	61.2 (58.2–64.2)
Malaysia	95.9 (94.9–97.0)	96.0 (95.0–97.1)	95.7 (94.6–96.8)	97.3 (96.5–98.2)	91.3 (89.7–92.8)	38.3 (35.6–41.0)
Myanmar	85.0 (81.0–89.1)	90.4 (87.0–93.7)	86.7 (82.9–90.1)	86.4 (82.5–90.2)	70.4 (65.3–75.6)	62.1 (56.6–67.6)
Nigeria	85.1 (80.1–90.0)	84.1 (79.0–89.1)	88.6 (84.7–93.0)	89.6 (85.3–93.8)	79.6 (74.0–85.2)	35.3 (28.7–41.9)
Philippines	88.8 (85.3–92.3)	81.5 (77.2–85.8)	93.0 (90.1–95.8)	93.9 (91.3–96.6)	81.5 (77.2–85.8)	34.2 (28.9–39.4)
Thailand	68.6 (66.7–70.4)	76.3 (74.6–78.0)	65.5 (63.5–67.4)	75.9 (74.2–77.6)	58.5 (56.5–60.5)	41.2 (39.2–43.2)
Turkey	80.2 (77.4–82.9)	81.3 (78.6–83.9)	82.2 (79.6–84.8)	79.7 (76.9–82.4)	78.4 (75.5–81.2)	74.7 (71.8–77.7)
Vietnam	88.3 (85.9–90.8)	89.9 (87.5–92.2)	48.2 (44.4–52.1)	69.3 (65.7–72.8)	16.0 (13.2–18.8)	86.0 (83.4–88.7)
Other	69.0 (62.1–75.8)	69.5 (62.7–76.4)	57.5 (50.1–64.8)	71.8 (65.2–78.5)	43.1 (35.7–50.5)	52.3 (44.9–59.7)

Other countries include Canada, China, Germany, India, Ireland, New Zeeland, Scotland, Sri-Lanka, and the United Kingdom.

As shown in Table 4, the levels of perceived COVID-19 vaccine effectiveness, acceptance, drivers of vaccine uptake decision-making, and vaccine uptake hesitancy remained significantly associated with the country of residence after controlling for socio-demographic and economic characteristics. For example, participants from Australia, Bangladesh, Indonesia, Iraq, Malaysia, Myanmar, the Philippines, Turkey, and Vietnam were significantly more likely to agree that vaccination would effectively control and prevent COVID-19 than participants from other countries. Compared to participants from other countries, participants from Bangladesh, Iraq, Malaysia, Myanmar, Thailand, Turkey, and Vietnam were significantly more likely to get the COVID-19 vaccines. Regarding hesitancy in vaccine uptake, participants from Austria, Indonesia, Iraq, Turkey, and Vietnam were significantly more likely to express their hesitancy to receive COVID-19 vaccines than participants from other countries.

**Table 4:** Factors related to perceived COVID-19 vaccine effectiveness, acceptance, and drivers of the uptake decision-making (*n* = 10477).

	Agreed that vaccines are effective to prevent and control COVID-19	Would accept COVID-19 vaccines when available	Believed that convenience is important for people to decide whether to accept vaccines	Believed that healthcare providers’ advice is important for people to decide whether to accept vaccines	Believed that cost of the vaccines is important for people to decide whether to accept vaccines	Expressed hesitancy to receive COVID-19 vaccines
**Country**						
Australia	2.83 (1.64–4.88)**	1.16 (0.72–1.86)	2.12 (1.32–3.38)**	2.05 (1.22–3.44)**	1.93 (1.23–3.03)**	1.01 (0.65–1.57)
Austria	1.31 (0.92–1.85)	1.30 (0.91–1.86)	0.80 (0.58–1.11)	0.81 (0.56–1.16)	0.40 (0.28–0.56)**	1.78 (1.29–2.45)**
Bangladesh	1.67 (1.08–2.58)*	4.81 (2.78–8.31)**	5.52 (3.43–8.89)**	3.02 (1.78–5.11)**	2.64 (1.78–3.91)**	0.98 (0.68–1.42)
Egypt	0.67 (0.47–0.96)*	1.34 (.92–1.95)	3.27 (2.29–4.69)**	2.06 (1.39–3.05)**	3.37 (2.39–4.76)**	0.38 (0.27–0.53)**
Indonesia	3.58 (2.35–5.45)**	1.47 (0.99–2.17)	1.26 (0.87–1.81)	6.61 (4.04–10.83)**	0.65 (0.45–0.94)*	2.17 (1.51–3.11)**
Iraq	2.74 (1.88–3.99)**	6.65 (4.42–10.02)**	2.83 (2.00–4.00)**	3.19 (2.15–4.73)**	0.64 (0.46–0.90)**	1.48 (1.06–2.06)*
Malaysia	7.57 (4.88–11.74)**	7.62 (4.88–11.89)**	10.68 (7.04–16.19)**	8.99 (5.51–14.66)**	8.25 (5.69–11.97)**	0.52 (0.37–0.72)**
Myanmar	1.91 (1.20–3.02)**	2.67 (1.60–4.45)**	4.05 (2.57–6.38)**	1.82 (1.13–2.93)*	3.10 (2.08–4.63)**	1.32 (0.90–1.94)
Nigeria	1.66 (0.99–2.78)	1.37 (0.82–2.30)	4.69 (2.74–8.01)**	2.21 (1.25–3.93)**	5.09 (3.19–8.13)**	0.44 (0.29–0.67)**
Philippines	3.11 (1.90–5.09)**	1.40 (0.89–2.21)	6.77 (3.95–11.61)**	3.33 (1.85–5.98)**	3.35 (2.18–5.15)**	0.46 (0.32–0.69)**
Thailand	1.05 (0.75–1.48)	1.62 (1.14–2.30)**	1.26 (0.92–1.74)	1.31 (0.92–1.87)	1.49 (1.08–2.05)*	0.58 (0.43–0.80)**
Turkey	2.16 (1.48–3.17)**	2.54 (1.72–3.75)**	3.45 (2.40–4.96)**	1.49 (1.00–2.20)*	4.58 (3.21–6.55)**	2.82 (1.99–3.98)**
Vietnam	3.33 (2.21–5.02)**	4.03 (2.64–6.17)**	0.60 (0.43–0.85)**	0.77 (0.52–1.13)	0.19 (0.13–0.28)**	5.54 (3.80–8.07)**
Other countries	R	R	R	R	R	R
**Age group**						
<30 years	R	R	R	R	R	R
31 to 45 years	0.66 (0.57–0.76)**	0.58 (0.50–0.68)**	0.66 (0.57–0.75)**	0.54 (0.46–0.63)**	0.77 (0.68–0.88)**	0.93 (0.82–1.04)
46 to 60 years	0.61 (0.51–0.71)**	0.54 (0.45–0.64)**	0.66 (0.57–0.77)**	0.49 (0.41–0.59)**	0.71 (0.61–0.82)**	0.95 (0.82–1.09)
60+ years	0.82 (0.65–1.03)	0.74 (0.57–0.94)*	0.85 (0.68–1.06)	0.86 (0.66–1.11)	0.62 (0.50–0.76)**	1.15 (0.94–1.40)
**Sex**						
Female	R	R	R	R	R	R
Male	1.28 (1.15–1.43)**	1.16 (1.03–1.30)*	1.09 (0.99–1.21)	1.09 (0.97–1.22)	0.99 (0.90–1.09)	1.21 (1.11–1.32)**
**Residential area**						
Rural	R	R	R	R	R	R
Urban	1.35 (1.21–1.51)**	1.40 (1.25–1.57)**	1.25 (1.12–1.39)**	1.17 (1.04–1.32)**	1.05 (0.95–1.17)	1.04 (0.95–1.15)
**Education level**						
< Secondary	R	R	R	R	R	R
Secondary	1.53 (1.33–1.76)**	1.42 (1.23–1.64)**	1.37 (1.20–1.56)**	1.08 (0.93–1.25)	1.35 (1.19–1.54)**	1.12 (0.99–1.27)
> Secondary	1.85 (1.61–2.13)**	2.68 (2.31–3.11)**	1.55 (1.36–1.77)**	2.31 (2.00–2.67)**	1.41 (1.23–1.61)**	1.18 (1.04–1.33)**
**Employment status**						
Employed	R	R	R	R	R	R
Unemployed/student	1.06 (0.92–1.21)	0.96 (0.83–1.11)	1.00 (0.88–1.14)	1.23 (1.06–1.42)**	1.25 (1.10–1.40)**	0.81 (0.72–0.90)**
**Marital status**						
Ever married	R	R	R	R	R	R
Never married	0.92 (0.82–1.03)	0.87 (0.77–0.99)*	1.14 (1.01–1.28)*	0.81 (0.72–0.93)**	1.05 (0.94–1.17)	1.12 (1.01–1.24)*
**Perceived family economic status**						
Low	R	R	R	R	R	R
Medium	1.17 (1.04–1.32)*	1.12 (0.99–1.27)	0.93 (0.83–1.05)	1.18 (1.04–1.35)*	0.76 (0.68–0.85)**	0.82 (0.74–0.90)**
High	1.46 (1.25–1.70)**	1.48 (1.25–1.75)**	0.81 (0.70–0.94)**	1.09 (0.93–1.28)	0.47 (0.41–0.54)**	0.90 (0.79–1.02)

Other countries include Canada, China, Germany, India, Ireland, New Zealand, Scotland, Sri-Lanka, and the United Kingdom. Data were presents as adjusted odds ratio (confidence interval). R: Reference

*P<0.05

**P<0.01.

The levels of perceived COVID-19 vaccine effectiveness, acceptance, drivers of vaccine uptake decision-making, and vaccine uptake hesitancy also remained significantly associated with socio-demographic and economic characteristics. For example, participants in the age groups of 31–45 years and 46–60 years were significantly less likely to agree that vaccination would effectively control and prevent COVID-19, accept the vaccines, and agree that convenience, health providers’ advice, and vaccine costs are important for deciding whether they would receive the vaccines relative to those who were 30 years or younger. Male participants were significantly more likely to agree that vaccination would effectively prevent and control COVID-19, accept the vaccines, and express their hesitancy to receive COVID-19 vaccines than female participants.

Urban participants were significantly more likely to agree that vaccination would effectively prevent and control COVID-19, accept the vaccines, and agree that convenience and health providers’ advice is important for deciding whether they would receive the vaccines. Compared to participants with education lower than secondary, participants with education higher than secondary level were significantly more likely to agree that vaccination would effectively prevent and control COVID-19; accept the vaccines; agree that convenience, health providers’ advice, and vaccine costs are important for deciding whether they would receive the vaccines; and express their hesitancy to receive COVID-19 vaccines. Employed and never married participants were significantly more likely to express their hesitancy to receive COVID-19 vaccines than employed and ever married participants, respectively. Compared to low-economic families, participants from medium- and high-economic families were significantly more likely to agree that vaccination would effectively prevent and control COVID-19, vaccine acceptance, health providers’ advice is important, and less likely to agree that vaccine convenience, and costs are important for deciding whether they would receive the vaccines.

## Discussion

This multi-country study assessed the prevalence and related factors of perceived effectiveness, acceptance, and potential drivers of people’s decision-making in COVID-19 vaccine uptake among a large-scale population recruited from 20 countries across four continents involving different socioeconomic and cultural contexts. We found that 78.8% of the participants agreed that vaccination would effectively control and prevent COVID-19, and 81.8% would get the vaccines if they would become available in the future. The participants believed that convenience (73.3%), health providers’ advice (81.8%), and costs of vaccines (57.0%) are important for deciding whether they would receive the vaccines. Almost half (47.2%) expressed their hesitancy to be vaccinated. Levels of perceived effectiveness ranged from 67.8% in Egypt to 95.9% in Malaysia and acceptance from 64.7% in Australia to 96.0% in Malaysia. Perceived drivers of vaccination decision-making also varied by country, ranging from 49.7% in Austria to 95.7% in Malaysia for convenience, 16.0% in Vietnam to 91.3% in Malaysia for costs of vaccines, and 31.5% in Egypt to 86.0% in Vietnam for hesitancy in vaccine uptake.

The findings are comparable with other existing studies. China showed the highest level of tendency to take COVID-19 vaccines (91.3%), whereas the lowest level was found in Congo (27.7%) [28–30]. A survey conducted in nine LIMCs reported COVID-19 vaccine acceptance rates of 76.4% to 88.8% [31]. The rapid dissemination of COVID-19 related information and increased infections and deaths worldwide can explain the high rates of perceived COVID-19 vaccine effectiveness and acceptance. These evolvements may raise the consciousness and lead to positive attitudes regarding the COVID-19 vaccines. A recent study conducted in LIMCs found that COVID-19 vaccine acceptance was positively related to COVID-19 knowledge and worries and fears of the pandemic [32]. In this study, regression analyses showed that the levels of perceived COVID-19 vaccine effectiveness, acceptance, drivers of vaccine uptake decision-making, and vaccine uptake hesitancy are significantly associated with the country of residence and several other socio-demographic and economic characteristics.

Individuals in the age groups of 31 to 45 and 46 to 60 were significantly less likely to agree that vaccination would successfully control and prevent COVID-19 and get the vaccine, compared to those aged 30 years or younger. They were also significantly more likely to agree that convenience, health providers’ advice, and vaccine costs are essential for deciding whether they would receive the vaccines. The existing literature also suggests that age was negatively correlated with the COVID-19 vaccine acceptance [33,34]. Similarly, youngers exhibited greater odds of COVID-19 vaccine acceptance compared to participants with older age in nine LIMCs [31]. This is conceivable because younger people are more exposed to information and engaged on online platforms such as the internet and social media. Therefore, they are more likely to stay updated on the latest information about the COVID-19 [35]. In contrast, most previous studies reported that older adults were more likely to get the COVID-19 vaccines than those in younger age groups [36–45]. A few other studies did not find significant relationships between age and COVID-19 vaccine acceptance [30,46,47].

Males had greater odds of perceived COVID-19 vaccine effectiveness and acceptance than females. Males were also more likely to agree that vaccination convenience and health providers’ advice were essential drivers of decision-making in COVID-19 vaccine uptake. These findings affirm previous studies that also reported a similar direction of association in terms of COVID-19 vaccine acceptance [30,36,37,39,40,42,44,45,47–53]. In addition, lower odds of willingness to be vaccinated were reported in females compared to males in a multi-country study conducted in nine LMICs [31]. Likewise, a previous study found an inverse association that females were more likely to express acceptance of COVID-19 vaccine than males [41]. However, multiple studies exhibited no relationships between sex and COVID-19 vaccine acceptance [33,38,43,54].

Perceived COVID-19 vaccine effectiveness and acceptance rates were higher among participants residing in urban areas than rural residents. Urbans were also significantly more likely to agree that vaccination convenience and health providers’ advice were essential drivers of decision-making in COVID-19 vaccine uptake. These findings corroborate previous studies that revealed the relationship between the increased willingness to take or accept COVID-19 vaccines among urban residents [39,42,51]. This association may be because urbans have a better COVID-19 prevention awareness than rural [55]. In contrast, another study found that participants from rural areas were more likely to express their tendency to receive COVID-19 vaccines than those in urban areas [31,33]. However, other studies did not find significant relationships [30,40,45].

Highly educated participants were more likely to perceive that COVID-19 vaccines would effectively control and prevent COVID-19 and accept them once available than those with lower education. In addition, they were more likely to believe that convenience, health providers’ advice, and vaccination costs are important drivers of people’s decision-making to receive the COVID-19 vaccines and express less hesitancy in its uptake. The findings are in line with previous studies showing that higher education is related to increased COVID-19 vaccine acceptance [33,42,56]. Similarly, another study in multiple LIMCs reported a positive relationship between education and COVID-19 vaccine acceptance [32]. Raising COVID-19 knowledge, particularly among lower-educated people, can effectively increase vaccine uptake [31]. However, this association was not detected in other studies [30,40,41,43,53,54].

Student or unemployed participants were more likely to believe that health providers’ advice and vaccination costs are important drivers of people’s decision-making to get the COVID-19 vaccines than employed participants. However, student or unemployed participants were less likely to express their hesitancy in the vaccine uptake. A study in England reported a positive association between employment and COVID-19 vaccine acceptance [46]. The relationship between education level and employment status can explain this finding. Employed people are more likely to have higher education and be aware of the COVID-19 pandemic and its prevention measures, including vaccination [57,58]. In contrast, a study in Israel found that unemployment was positively related to willingness to get COVID-19 vaccines and negatively associated with inoculation [59]. Other studies did not find relation between employment status and acceptance of COVID-19 vaccine [30,40,41,43,54].

In this study, never married respondents were more likely to believe that convenience is a crucial determinant of people’s decision-making to get the COVID-19 vaccines; whereas, ever married participants believed the healthcare workers’ advice. Never-married respondents were less likely to have vaccine acceptance, and more likely to have hesitancy in the vaccine uptake than ever-married respondents. This finding may indicate greater concern about COVID-19 among unmarried people. It may also be explained by the higher exposure to COVID-19 related information among younger groups as they tend to be more active in accessing information and social media [35]. This direction of the relation is in line with a previous study in Uganda [47] but inconsistent with other studies that reported married participants were more likely to express their intention to get the COVID-19 vaccines [30,38,54]. Some other studies did not find relation between marital status and acceptance of COVID-19 vaccine [40,53]

Participants from medium- and high-economic families were significantly more likely to agree that vaccination would effectively prevent and control COVID-19than participants from low-economic families. They were less likely to agree that vaccine costs are important for deciding whether to receive the vaccines. The reduced COVID-19 vaccines acceptance rates among the low-income group may be due to scarcity of high-quality information and limited health literacy among lower-income individuals [60]. The present findings are partially supported by other studies that reported that income was positively related to the acceptance of the COVID-19 vaccines [40,42,46]. In contrast, a study in China did not find this relationship [30,40].

This multi-country survey is among a few studies exploring factors that may contribute to COVID-19 vaccine uptake improvement using extensive data collected from populations in countries with different socioeconomic and cultural contexts. However, this study has several limitations. Response biases could be one of the significant limitations because data were self-reported and collected through online platforms. The snowball technique used to recruit respondents could hamper the heterogeneity in the study sample. Another significant limitation is the study population representativeness that included a higher proportion of highly educated and urban samples more likely to access the online surveys. Moreover, the present study included the limited measures as it didn’t investigate any health literacy-related information.

In conclusion, most of the general adult population in this multi-country study believed vaccination would effectively control and prevent COVID-19. They would accept the vaccines when they become available. Perceived drivers of people’s vaccination decision-making included convenience, health providers’ advice, and vaccination costs. About half had been vaccinated or would accept the vaccination when the vaccines become available, while the other half expressed their hesitancy to be vaccinated. Several economic and socio-demographic factors are related to the perceived COVID-19 vaccine effectiveness, acceptance, and hesitancy in the populations. These factors are critical and should be considered as essential elements in developing COVID-19 vaccination programs to boost the vaccine uptake in the populations.
